# Pediatric-Onset Multiple Sclerosis in the Gulf: A Scoping Review of the Current Evidence Base and Research Gaps

**DOI:** 10.3390/biomedicines14102195

**Published:** 2026-09-28

**Authors:** Eslam Shosha, Najeeb Qadi, Rakan AlRomayan, Musaad AbuKhalid

**Affiliations:** 1 Neuroscience Centre of Excellence, King Faisal Specialist Hospital and Research Centre, MBC 76, Riyadh 11211, Saudi Arabia; 2 Department of Medicine, Division of Neurology, McMaster University, Hamilton, ON L8S 4L8, Canada; 3 College of Medicine, Alfaisal University, Riyadh 11533, Saudi Arabia; 4 College of Medicine, King Saud University, Riyadh 11461, Saudi Arabia

**Keywords:** pediatric multiple sclerosis, children, Gulf Cooperation Council, scoping review, epidemiology, demyelinating disease, Middle East

## Abstract

**Background:** MS begins before age 18 in 3–10% of cases (pediatric-onset MS, POMS). Adult MS epidemiology is increasingly characterized across the Gulf, but whether the pediatric literature is sufficiently mature and ho0m1ogeneous to support a quantitative meta-analysis, as opposed to a scoping review, remains unclear. **Objective:** To map published POMS evidence across the six Gulf Cooperation Council (GCC) states and identify evidence gaps. **Methods:** A scoping review followed the Arksey and O’Malley framework, reported per PRISMA-ScR. Eligibility followed a Population-Concept-Context framework: MS onset before 18 years; epidemiological, clinical, diagnostic, therapeutic, and outcome data; the six GCC states. Adult-inclusive studies that did not report a dedicated pediatric-onset subgroup were retained only as contextual evidence and were not counted as meeting the Population criterion (see Methods). MEDLINE/PubMed, Embase, Scopus, Web of Science, and IMEMR were searched from inception through August 2026. Results: Eleven studies were included. Dedicated, pediatric-specific POMS data were identified from three of six countries: Kuwait (three epidemiological/clinical registry studies and two pediatric monoclonal-antibody outcome studies), Saudi Arabia (one national-registry study), and the UAE (two hospital/multicenter cohorts and one pediatric monoclonal-antibody outcome study). Qatar and Oman contributed only adult-inclusive prevalence studies without a dedicated pediatric subgroup, and no eligible study was identified from Bahrain. Incidence ranged from 2.1/100,000 (Kuwait) to a UAE-reported 7.2/100,000 in a 15–19-year age band that extends beyond the review’s < 18-year POMS definition; prevalence (2.7–36/100,000) was highest in the UAE. A relapsing–remitting course, female preponderance, and brainstem/cerebellar or visual presentations predominated; MRI, cognitive, and quality-of-life data were sparse or absent. Three studies reported pediatric natalizumab/ocrelizumab outcomes (Kuwait, UAE). **Conclusion:** The Gulf POMS evidence base is real but narrow, concentrated, and heterogeneous; a scoping review, which maps the evidence base and treats geographic and domain gaps as findings in their own right, is currently the more informative synthesis than a quantitative meta-analysis. This supports a harmonized, multi-country Gulf pediatric MS registry with standardized outcome reporting, including neurocognitive and quality-of-life measures.

## 1. Introduction

Multiple sclerosis (MS) is a chronic, immune-mediated, demyelinating disease of the central nervous system that most commonly presents in young adulthood. However, an estimated 3–10% of all MS cases begin before the age of 18 years, a presentation defined as pediatric-onset multiple sclerosis (POMS) [1,2,3]. Compared with adult-onset disease, POMS is characterized by a higher relapse frequency in the early years, a predominantly relapsing-remitting course at onset, and because disability accumulates from a much younger baseline, the potential for substantial lifetime impact on education, neurocognitive development, employment, and psychosocial functioning [2,3].

Epidemiological and clinical data on adult-onset MS across the Gulf region have expanded considerably over the past two decades, driven largely by the establishment of national MS registries in Kuwait and Saudi Arabia and by growing neuroimmunology capacity across the Gulf Cooperation Council (GCC) states [4,5,6]. In this review, “the Gulf” is used, consistent with prior regional epidemiological syntheses, as a neutral shorthand for the six GCC member states; the adjoining body of water is referred to throughout this manuscript as the Arabian Gulf. A fuller operational definition is given in Section 2.2. A regional systematic review and meta-analysis of MS incidence and prevalence across the Arabian Gulf area spanning Bahrain, Kuwait, Oman, Qatar, Saudi Arabia, and the UAE confirmed a marked and ongoing rise in reported incidence and prevalence, attributed in part to improved case ascertainment, wider MRI access, and changing environmental and lifestyle exposures [6]. That review, however, pooled adult and pediatric cases and did not isolate the pediatric subgroup [6].

Pediatric MS epidemiology lags behind the adult literature both globally and regionally. A global systematic review reported wide variation in POMS incidence (0.05–2.85 per 100,000) and prevalence (0.69–26.92 per 100,000) across the relatively small number of countries with dedicated data, and explicitly noted that data from the Middle East remained limited [7]. Within the Gulf specifically, individual registry- and hospital-based studies have since been published from Kuwait, Saudi Arabia, and the UAE [8,9,10,11,12,13], but to our knowledge no synthesis has yet mapped this literature as a distinct regional evidence base for pediatric disease.

Before undertaking any formal evidence synthesis, it is necessary to establish whether the existing literature is sufficiently voluminous, homogeneous, and complete in its geographic coverage to support quantitative meta-analytic pooling, or whether a scoping review—which maps the extent and nature of evidence without requiring homogeneous outcome reporting or a pooled effect estimate—is the more appropriate and informative approach [14,15]. We note that systematic reviews do not necessarily entail meta-analysis, and are not methodologically opposed to scoping reviews; the relevant question is not “scoping review versus systematic review” as such, but whether the available evidence can support meaningful quantitative pooling. A scoping review was chosen because the objective of this work is to map the extent, range, and gaps of the evidence rather than to estimate a pooled effect. The literature identified in this review (Section 3) comprises a small number of primary studies concentrated in fewer than half of the region’s countries, with heterogeneous study designs and outcome reporting, and with several countries apparently unrepresented in the dedicated POMS literature despite established adult MS surveillance infrastructure. These features are more consistent with a scoping review than quantitative meta-analytic pooling; this corroborates, rather than determines, the choice of design.

### Objectives and Review Questions

The objective of this scoping review is to map the extent, range, and nature of published evidence on POMS across the Gulf region and to identify evidence gaps to guide future primary research, regional collaboration, and clinical guidance. Specifically, this review addresses:

**RQ1.**  *What is the extent and geographic distribution of published literature on POMS across GCC countries?*

**RQ2.**  *What epidemiological, clinical, diagnostic, therapeutic, and patient-reported outcome data have been reported?*

**RQ3.**  *What are the key evidence gaps, by country and by domain?*

**RQ4.**  *What future research priorities and opportunities for regional collaboration are suggested by the mapped evidence gaps?*

## 2. Methods

### 2.1. Protocol and Reporting

This scoping review was conducted using the methodological framework of Arksey and O’Malley [14], as refined by Levac and colleagues [15], and drawing on the Joanna Briggs Institute (JBI) methodology for scoping reviews [16]. Reporting follows the PRISMA Extension for Scoping Reviews (PRISMA-ScR) checklist [17], the applicable PRISMA reporting standard for this study design; a completed PRISMA-ScR checklist, indicating the manuscript location of each item, is provided in the Supplementary Material. The corresponding PRISMA-ScR flow diagram of study identification and selection is presented in Figure 1. No formal protocol registration was undertaken for this review; scoping reviews are not eligible for registration on PROSPERO, and the authors elected not to register the protocol elsewhere. This is reported in accordance with PRISMA-ScR Item 5, which permits, but does not require, protocol registration.

### 2.2. Eligibility Criteria (Population-Concept-Context)

Population: Children and adolescents with onset of neurological symptoms or diagnosis of MS before 18 years of age, consistent with International Pediatric Multiple Sclerosis Study Group (IPMSSG) criteria [18].

Concept: Any aspect of POMS, including incidence/prevalence, demographic and clinical characteristics, diagnostic work-up (MRI, cerebrospinal fluid, evoked potentials), differential diagnosis from other acquired demyelinating syndromes, disease course and prognosis, disease-modifying therapy (DMT) use and outcomes, relapse management, cognitive function, quality of life, caregiver/family burden, and biomarker or genetic association studies.

Context: The Gulf region, operationalized as the six GCC member states (Saudi Arabia, UAE, Kuwait, Qatar, Oman, and Bahrain), consistent with prior regional epidemiological syntheses that group these countries together [6]. These six states border the Arabian Gulf. We use the term “Gulf” throughout to refer to this group of littoral states. A map showing the location of the six GCC states is provided in Figure 2. Studies of adult MS cohorts that did not report a dedicated pediatric-onset subgroup or age-stratified pediatric data were not considered to meet this Population criterion. Where such adult-inclusive studies constituted the only country-level source identified (Qatar, Oman; Section 3.2), they were retained in the review as contextual evidence documenting the absence of dedicated pediatric-specific reporting, rather than as formally included POMS-specific sources.

Eligible source types were peer-reviewed original research (registry-based cohorts, cross-sectional studies, case–control studies, and case series of five or more patients, as smaller series provide limited clinical information and cannot support meaningful description of clinical patterns or estimates of frequency), conference abstracts with extractable quantitative data, and clinical practice guidelines or consensus statements containing a dedicated pediatric section. Single case reports were excluded from formal data charting but were noted narratively where they represented the only identified evidence for a given country. Reviews, editorials, and non-English full texts without an available English abstract were excluded from data extraction but screened for relevant citations (snowballing). No lower date limit was applied, given the small overall volume of literature identified.

### 2.3. Information Sources and Search Strategy

MEDLINE/PubMed, Embase, Scopus, Web of Science Core Collection, and IMEMR were searched from inception to 9 August 2026, using the following search terms: (“multiple sclerosis” OR “demyelinating disease*” OR “disseminated sclerosis”) AND (child* OR pediatric* OR paediatric* OR adolescen* OR juvenile* OR childhood OR “early onset” OR “early-onset” OR “pediatric-onset” OR “paediatric-onset” OR “childhood-onset” OR “childhood onset”) AND (Gulf OR “Arabian Gulf” OR “Persian Gulf” OR GCC OR “Saudi Arabia” OR “United Arab Emirates” OR UAE OR Kuwait OR Kuwaiti OR Qatar OR Qatari OR Oman OR Omani OR Bahrain OR Bahraini). Reference lists of all included studies and relevant prior reviews were hand-searched for additional citations (backward and forward snowballing). Google Scholar was used as a supplementary source for citation searching only. MENACTRIMS and ECTRIMS conference abstract archives were also searched for relevant conference literature.

We recognize that requiring pediatric or age-related terminology in the search string could miss general or adult-inclusive GCC MS registry studies that report extractable pediatric-subgroup data without using pediatric terminology in the title or abstract. Given the review’s AND-structured search logic (MS terms AND pediatric terms AND Gulf/country terms), such studies would not be retrieved by the primary database search and could only be captured through the backward and forward citation snowballing described above, which is consistent with how the Qatar and Oman sources discussed in Section 3.2 were most likely identified.

### 2.4. Data Charting Process and Data Items

A standardized, piloted data-charting form was used to extract: first author and year; country and city/center; study design; data source (national registry, single-center, multicenter); study period; sample size; the definition of pediatric onset and diagnostic criteria applied (e.g., 2013 IPMSSG revision versus earlier definitions, 2010 or 2017 McDonald criteria) [18,19]; sex distribution; incidence/prevalence estimates and denominators; presenting clinical features; MRI and cerebrospinal fluid findings; treatment data; disease course and disability outcomes; and funding/conflict-of-interest statements. Data extraction was performed independently by two reviewers (M.A. and N.Q.), with discrepancies resolved through consensus discussion.

Six evidence domains were defined a priori to structure data charting and the subsequent evidence-gap synthesis (Section 2.5): (1) epidemiology (incidence/prevalence); (2) clinical phenotype; (3) diagnostic and MRI/imaging characterization; (4) disease-modifying therapy and treatment outcomes; (5) cognitive and quality-of-life outcomes; and (6) biomarker/genetic associations. These domains structure the evidence-gap map presented in Table 1.

### 2.5. Synthesis of Results

Findings were synthesized narratively and tabulated by country and by the six a priori evidence domains defined in Section 2.4 (Table 1), consistent with scoping review methodology. For this gap map, a domain was classified as a dedicated evidence gap for a given country (– in Table 1) when no peer-reviewed, pediatric-specific data addressing that domain were identified for that country; a domain was classified as partial evidence (±) when pediatric-relevant data existed only as an embedded subgroup within a predominantly adult cohort, or within a non-pediatric-specific study that incidentally included some pediatric-onset cases. In this framing, the future research priorities in RQ4 correspond to the domains and countries classified as – or ±, and the recommendation for a harmonized regional registry (Section 4) is consistent with dedicated evidence being concentrated in jurisdictions with pre-existing national MS registries; no formal consensus or prioritization method (for example, a Delphi process) was used, and these priorities are a descriptive reading of the gap map rather than formal recommendations. Consistent with scoping review methodology as described by the JBI [16] and PRISMA-ScR guidance [17], no formal risk-of-bias or methodological quality appraisal was performed; this review maps what has been published rather than evaluating the certainty of specific effect estimates, and results should be interpreted accordingly.

## 3. Results

### 3.1. Study Selection

The systematic search across MEDLINE/PubMed (*n* = 293), Scopus (*n* = 54), Web of Science (*n* = 138), Embase (*n* = 112), and IMEMR (*n* = 48) identified 645 records. After deduplication (*n* = 119), 526 records were screened at the title and abstract level. Of these, 490 were excluded based on eligibility criteria, leaving 36 reports sought for full-text retrieval. Following full-text assessment, 27 reports were excluded: 25 did not meet eligibility criteria (including adult-only populations, non-Gulf settings, and non-original research), and 2 were duplicate reports discovered during full-text assessment. Citation searching identified 7 additional reports, of which 2 met eligibility criteria. A total of 11 studies are presented in this review (Table 2): nine provided dedicated, pediatric-specific POMS data meeting the full Population-Concept-Context eligibility criteria (five from Kuwait, one from Saudi Arabia, three from the UAE), and two were adult-inclusive prevalence studies from Qatar and Oman that lacked a dedicated pediatric subgroup; these two are retained as contextual evidence documenting the absence of dedicated pediatric-specific reporting in those countries, rather than as formally included POMS-specific sources (Section 2.2). This total is consistent with the screening counts shown in Figure 1 (9 database-identified + 2 citation-identified = 11). The PRISMA-ScR flow diagram is presented in Figure 1.

### 3.2. Geographic and Temporal Distribution of Evidence

Of the six target countries, dedicated pediatric-specific POMS data meeting the full eligibility criteria were identified for three countries: Kuwait, Saudi Arabia, and the UAE (Table 2). All five Kuwaiti publications identified [8,9,10,20,21]—three epidemiological/clinical reports and two pediatric monoclonal-antibody outcome studies—draw on the same National MS Registry, established in 2011 with retrospective data extending back to 1994; they therefore represent a single underlying registry population captured at different time points and for different outcome domains, rather than independent cohorts, and should not be interpreted as replication across separate populations. Saudi Arabia contributed one national-registry study drawing on the National Saudi MS Registry (operational 2015–2018) [11]. The UAE contributed two hospital/multicenter epidemiological cohorts based in Abu Dhabi [12,13], and one further pediatric monoclonal-antibody outcome study [22] drawing on two of the same Abu Dhabi centers; these UAE sources likewise overlap substantially in their underlying patient populations rather than representing fully independent samples. For Qatar, the only identifiable primary source (Deleu et al., 2013) is an adult-inclusive MS prevalence study that reports no dedicated pediatric or early-onset subgroup analysis; a previously cited “5% early-onset subgroup” figure could not be verified against that source [23], and this study does not meet the Population criterion for formal inclusion as POMS-specific evidence (Section 2.2). For Oman, similarly, the only identifiable primary source (Al-Senani et al., 2021) is a hospital-based, adult-inclusive MS prevalence study (Sultan Qaboos University Hospital and Khoula Hospital, 2006–2019) reporting a crude prevalence of 15.9 per 100,000, an incidence of 1.38 per 100,000 (2015–2018), and a mean age at onset of 27.3 ± 7.7 years; no dedicated pediatric or early-onset subgroup analysis was reported [24], and this study is likewise retained only as contextual evidence of the absence of dedicated pediatric reporting rather than as a formally included POMS-specific source. No eligible POMS-specific peer-reviewed studies were identified from Bahrain, despite it also contributing to the general adult MS epidemiology literature captured in regional reviews [6].

### 3.3. Epidemiology: Incidence and Prevalence

Reported incidence and prevalence estimates varied by country, population denominator (national registry vs. hospital catchment vs. Emirati-national-only), and study period. In Kuwait, POMS incidence was 2.1 per 100,000 and prevalence 6.0 per 100,000 in 2013, with statistically significant annual increases in both incidence (approximately 5%) and prevalence (approximately 6%) over 1994–2013 [8]. In Saudi Arabia, hospital-registry prevalence was 2.73 per 100,000, with a nationally projected prevalence of 14.33 per 100,000 pediatric population [11]. In the UAE, among Abu Dhabi Emirati nationals, age- and sex-adjusted prevalence was 26 per 100,000 in males and 36 per 100,000 in females, and incidence rose from 2.3 per 100,000 in the 10–14-year age band to 7.2 per 100,000 in the 15–19-year band; because this older band spans ages 18–19 and therefore extends beyond this review’s < 18-year POMS definition, the 7.2 per 100,000 figure should not be interpreted as an unqualified POMS-specific incidence estimate, and is presented here descriptively as reported by the original study [12]. The original authors noted these UAE figures were higher than most international comparator cohorts. No population-based incidence or prevalence estimates specific to POMS (i.e., restricted to onset before age 18) were identified from Qatar, Oman, or Bahrain.

### 3.4. Demographic and Clinical Characteristics

A consistent female preponderance was reported across all cohorts, with a female-to-male ratio of approximately 2.4:1 in the largest Kuwaiti cohort [10] and female proportions of 65–74% in the UAE and Saudi cohorts [11,12,13]. Mean age at onset clustered narrowly around 14–15.7 years across all cohorts (Kuwait ~15.1 years [10]; Saudi Arabia 15.7 years [11]; UAE 15.0 years, with 50.6% of onsets before age 16 [13]).

Presenting symptoms were broadly comparable across cohorts. In the largest Kuwaiti cohort, brainstem/cerebellar (34.9%), spinal (29.3%), and visual pathway (27.3%) symptoms were the most frequent presenting features [10]. In the UAE cohort, onset before age 12 was characterized predominantly by visual symptoms, while onset at or after age 12 presented with a broader mixture of visual, motor, and sensory features [12].

A relapsing-remitting course predominated at last follow-up across cohorts (83.5% in the 2024 Kuwaiti cohort [10]).

### 3.5. Diagnostic Approach

All identified dedicated pediatric-specific studies applied variants of the IPMSSG consensus criteria (2013 revision or the earlier 2007 definitions) [18] for case definition, with MRI and cerebrospinal fluid analysis used for diagnosis and differential exclusion of acute disseminated encephalomyelitis and neuromyelitis optica spectrum disorder in most cohorts; this statement does not extend to the adult-inclusive Qatar and Oman sources, which did not apply pediatric-specific diagnostic criteria. However, no dedicated, systematically reported MRI phenotyping study restricted to a Gulf pediatric population was identified; a related Emirati MRI characterization study of MS onset was not restricted to pediatric patients [25]. Granular lesion-distribution, spinal cord, and optic nerve imaging data specific to Gulf POMS cohorts therefore remain largely undescribed in the peer-reviewed literature.

### 3.6. Treatment and Disease-Modifying Therapy

Interferon beta-1a was reported as the most frequently used first-line disease-modifying therapy in the UAE cohort (48% of patients) [12]. The 2024 Kuwaiti cohort reported an annual relapse rate of 0.18 during the first two years of treatment; the specific agents driving this reported outcome were not detailed in the available abstract [10]. Three dedicated pediatric monoclonal-antibody outcome studies, all from the two countries with established national or multicenter cohorts, were identified. In Kuwait, a prospective national-registry study of natalizumab in 32 pediatric patients with active relapsing MS reported a significant reduction in annualized relapse rate (1.66 ± 0.5 to 0.06 ± 0.25; *p* < 0.001) and improvement in EDSS (3.3 ± 1.3 to 2.2 ± 1.0; *p* < 0.001) at last follow-up, with two-thirds of patients having had breakthrough disease on first-line therapy [20]. A subsequent Kuwaiti national-registry cohort of 24 pediatric patients treated with ocrelizumab reported EDSS stabilization in 75% and improvement in a further 16.7%, MRI activity in only 4.2%, and no evidence of disease activity (NEDA-3) in 91.7% [21]. In the UAE, a two-center retrospective review (Cleveland Clinic Abu Dhabi and Sheikh Shakhbout Medical City) of 29 pediatric patients treated with ocrelizumab (97% relapsing-remitting at treatment initiation) reported safety and tolerability outcomes broadly consistent with those described in the international POMS and adult MS ocrelizumab literature [22]. As uncontrolled observational cohorts without formal risk-of-bias appraisal (Section 2.5), the natalizumab and ocrelizumab findings summarized above are best interpreted as descriptive reported outcomes from the identified registries rather than as confirmatory evidence of comparative safety or effectiveness. These three studies represent the first dedicated pediatric monoclonal-antibody outcome data identified for the region; outcome data specific to oral disease-modifying therapies (e.g., fingolimod, teriflunomide, or dimethyl fumarate) in Gulf pediatric cohorts remain unreported, despite international randomized trial data supporting pediatric use of several of these agents [26,27]. The Saudi national consensus recommendations include a dedicated pediatric and adolescent MS management section, but this represents expert consensus rather than regional outcomes data [28].

### 3.7. Patient-Reported, Cognitive, and Quality-of-Life Outcomes

No study addressing cognitive functioning, educational attainment or impact, psychosocial burden, caregiver/family burden, or health-related quality of life in a Gulf pediatric MS population was identified in this review. This represents a notable gap given the increasing recognition, in the international literature, of cognitive impairment and quality-of-life burden as central and under-recognized dimensions of POMS morbidity [29].

### 3.8. Evidence Gap Map

Table 1 summarizes the distribution of available evidence across the six target countries and six evidence domains identified a priori (Section 2.4). Dedicated, country-specific evidence was most complete for basic epidemiology and clinical phenotype in Kuwait, Saudi Arabia, and the UAE, and Kuwait and the UAE now also have dedicated pediatric-specific DMT outcome data following the addition of the natalizumab and ocrelizumab studies discussed in Section 3.6. Evidence remained essentially absent across all domains for Bahrain, and largely absent beyond adult-inclusive, non-pediatric-specific prevalence data for Qatar and Oman. Consistent with Section 3.2, the Qatar and Oman entries in Table 1 reflect the absence of dedicated pediatric-specific evidence within adult-inclusive registries, rather than a null finding from a dedicated pediatric study. No country had dedicated data on cognitive or quality-of-life outcomes, or on biomarker/genetic associations, specific to a pediatric Gulf cohort.

## 4. Discussion

This scoping review mapped a real but narrow evidence base on POMS across the Gulf region. Dedicated pediatric data exist for only three of six target countries, and that evidence is driven disproportionately by two national registries (Kuwait, Saudi Arabia) and a small number of hospital-based cohorts (UAE). Encouragingly, the Kuwaiti and UAE registries/cohorts have begun to generate dedicated pediatric therapeutic outcome data as well, with three recent studies [20,21,22] reporting natalizumab and ocrelizumab outcomes specifically in Gulf POMS cohorts, a domain that was previously entirely unrepresented in the regional literature. Qatar, Oman, and Bahrain-all of which maintain adult MS epidemiology literature and, in at least Qatar’s case, functioning tertiary neurology referral systems-remain essentially undocumented for pediatric-onset disease specifically. This pattern most plausibly reflects a publication and research-infrastructure gap rather than a true absence of pediatric cases, since POMS prevalence estimates elsewhere in the region imply that all six GCC states almost certainly manage some number of affected children.

Where data exist, the regional clinical picture broadly mirrors the international POMS literature: a consistent female preponderance, onset concentrated in later childhood and adolescence, brainstem/cerebellar, visual, and spinal presenting symptoms, and a predominantly relapsing-remitting course at onset [2,3,7]. Within the region, the UAE cohort further suggested an age-dependent clinical phenotype, with visual symptoms predominating before age 12 and a broader mixture of visual, motor, and sensory features thereafter [12]; if replicated, this observation would be consistent with age-related differences in presentation described in the broader POMS literature, in which younger children more often present with polyfocal or encephalopathic features while older adolescents present more similarly to adult-onset disease [2,3]. This concordance is reassuring for baseline clinical recognition and supports the applicability of international POMS diagnostic and management frameworks, such as IPMSSG criteria, to Gulf pediatric practice [18]. Two regional findings, however, warrant cautious interpretation rather than direct extrapolation: the comparatively high incidence and prevalence estimates reported in the Emirati-national UAE cohort. These findings derive from single-center or single-nationality cohorts subject to referral bias, and both spanned periods before and after key revisions to pediatric MS diagnostic criteria (the 2013 IPMSSG revision) and general MS diagnostic criteria (the 2010 and 2017 McDonald criteria) [18,19], which may affect case ascertainment and comparability across studies and over time.

These features-small absolute numbers of studies, concentration in fewer than half of the target countries, heterogeneous case ascertainment (national registry versus single hospital), diagnostic-era heterogeneity, and inconsistent outcome reporting-are the central reasons quantitative meta-analytic pooling is not currently appropriate for this topic, and a scoping review-rather than a systematic review with meta-analysis-is the more informative synthesis at this stage. Pooling incidence, prevalence, or clinical-outcome estimates across such a heterogeneous and geographically incomplete set of studies risks producing a spuriously precise regional estimate that is, in reality, driven by whichever two or three countries happen to have registries, while silently excluding the experience of countries without published data. A scoping review avoids this distortion: it maps what is known, explicitly documents what is not known, and unlike a systematic review treats the absence of data from Oman, Bahrain, and (for most domains) Qatar as a primary finding rather than as a limitation to be managed away.

The clinical and research implications follow directly from the gap map (Table 1). Clinically, pediatric neurologists in under-represented countries may be managing POMS without access to local or even regional benchmark data on expected disease course, treatment response, or the differential diagnostic yield of MRI and cerebrospinal fluid findings in their own population-a particular concern given that misclassification against acute disseminated encephalomyelitis or neuromyelitis optica spectrum disorder carries direct treatment consequences [18]. From a research standpoint, the most actionable next step is the extension of the Kuwaiti and Saudi national-registry model to a harmonized, multi-country Gulf pediatric MS registry, ideally coordinated through an existing regional platform such as the Middle East North Africa Committee for Treatment and Research in Multiple Sclerosis (MENACTRIMS) or GCC health-cooperation mechanisms. Such a registry would enable prospective, standardized capture of MRI phenotyping and disease-modifying therapy exposure and outcomes; monoclonal antibody data have begun to emerge from Kuwait and the UAE [20,21,22], but outcome data for newer oral agents now approved in pediatric populations elsewhere remain unreported regionally [26,27]. Critically, the cognitive, educational, and quality-of-life outcomes that are entirely absent from the current regional literature, despite their recognized centrality to POMS morbidity internationally, should also be prioritized [29].

Comparison with the European and North American POMS literature further situates the regional treatment findings. High-efficacy therapies (HETs, including natalizumab, ocrelizumab, and other B-cell-depleting or highly potent agents) have increasingly been used as first-line or early treatment in pediatric MS cohorts in Europe and North America [30,31], reflecting a broader shift toward early aggressive treatment strategies now supported by large multinational registry data showing reduced long-term disability accrual with early initiation of high-efficacy therapy in pediatric-onset MS [32,33], alongside recognition that early disease control may help protect neurocognitive and educational trajectories in this population [29]. Against this backdrop, the regional data identified in this review—an annual relapse rate of 0.18 in the largest Kuwaiti cohort during early treatment, and the natalizumab and ocrelizumab outcomes summarized in Section 3.6—are broadly consistent with the trajectory of increasing HET use described internationally, and suggest that Gulf pediatric neurology centers with established registries have begun to adopt similar treatment approaches. However, regional HET outcome data remain limited to two countries and two agents (natalizumab, ocrelizumab), with no regional data yet available on other HETs or on oral disease-modifying therapies with pediatric trial evidence (e.g., fingolimod, teriflunomide). Whether HET use, sequencing, and treatment thresholds in Gulf pediatric practice otherwise mirror European and North American approaches—including the increasingly early-HET treatment paradigm supported by recent registry evidence [30,31,32,33]—cannot be determined from the currently published regional literature and represents an important question for the proposed harmonized Gulf pediatric MS registry.

## 5. Limitations

Several limitations of this review should be acknowledged. First, although we conducted a systematic search across multiple databases including Embase and IMEMR, it is possible that relevant Arabic-language regional publications, unpublished registry reports, or conference abstracts were not captured, particularly for Qatar, Oman, and Bahrain, where the true absence of dedicated POMS studies cannot be fully confirmed. Relatedly, because our search strategy required pediatric or age-related terminology, we cannot fully exclude the possibility that adult-inclusive GCC MS registry publications containing unindexed pediatric-subgroup data were missed, despite the citation-snowballing mitigation described in Section 2.3. Second, as with any scoping review, no quality or risk-of-bias appraisal of included sources was performed; findings describe what has been published, not the methodological rigor or certainty of specific estimates, and should not be used to draw causal or precise effect-size conclusions. Third, the restriction to peer-reviewed literature may have excluded valuable grey literature sources, though we did search MENACTRIMS and ECTRIMS conference abstracts. Fourth, the heterogeneity in diagnostic criteria across studies (2007 vs. 2013 IPMSSG criteria, and 2010 vs. 2017 McDonald criteria) may affect comparability of findings across cohorts and over time. Finally, the small number of included studies and their geographic concentration limit the generalizability of our findings to the entire Gulf region.

## 6. Conclusions

Published evidence on pediatric-onset multiple sclerosis in the Gulf region is real but limited: dedicated data exist for only three of six target countries, driven largely by national registries in Kuwait and Saudi Arabia and hospital-based cohorts in the UAE, with Qatar and Oman contributing no dedicated pediatric-specific evidence—only adult-inclusive prevalence data without a dedicated pediatric subgroup and Bahrain entirely absent from the dedicated pediatric literature. Where present, findings are broadly consistent with international POMS patterns, but sample sizes, referral bias, and diagnostic-era heterogeneity limit generalizability. These characteristics indicate that the evidence base is not yet sufficiently mature or homogeneous to support quantitative meta-analytic pooling; a scoping review is the more appropriate and informative synthesis at this time, since it maps the evidence base comprehensively and treats geographic and domain-level gaps as substantive findings. The results support the establishment of a harmonized, multi-country Gulf pediatric MS registry with standardized diagnostic criteria and outcome reporting. Encouragingly, dedicated pediatric monoclonal-antibody outcome data have begun to emerge from Kuwait and the UAE; MRI phenotyping, oral disease-modifying therapy outcomes, and, in particular, the neurocognitive and quality-of-life measures that remain absent from the regional literature should be prioritized next.

## Figures and Tables

**Figure 1:**
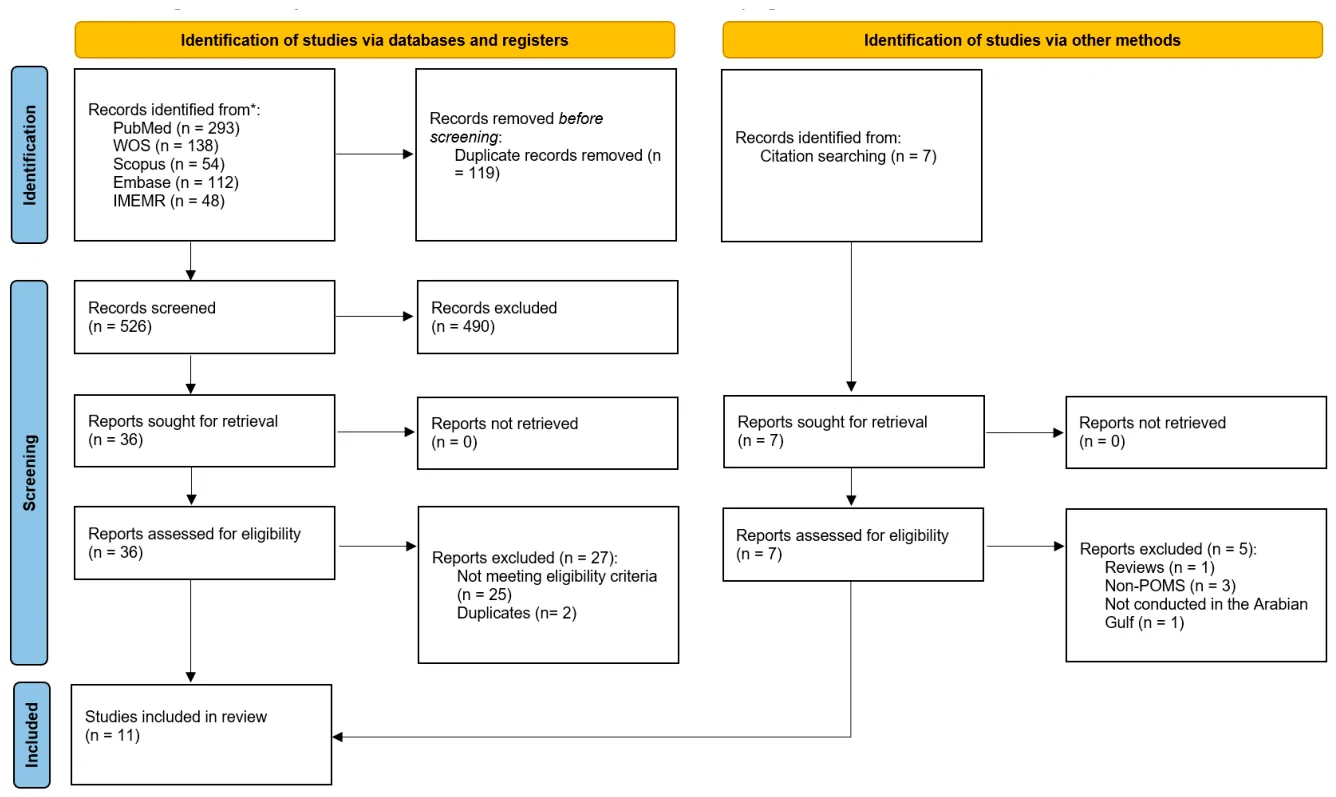
PRISMA-ScR flow diagram of study selection. * Searched database.

**Figure 2:**
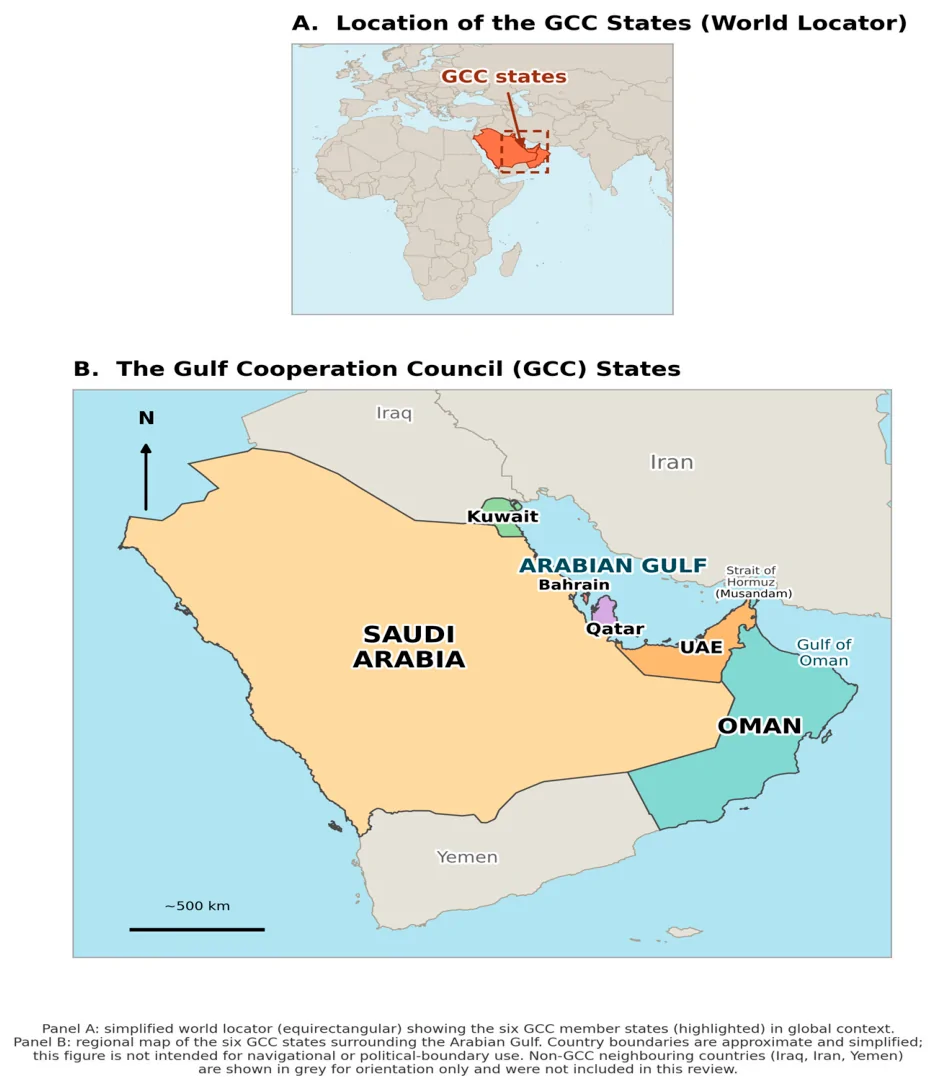
The GCC States and Their Location. The six Gulf Cooperation Council (GCC) member states—Saudi Arabia, Kuwait, Bahrain, Qatar, the United Arab Emirates (UAE), and Oman—that together comprise “the Gulf” as operationalized in this review (Section 2.2). Panel (**A**) shows the location of the GCC states in a global context. Panel (**B**) shows the GCC states surrounding the Arabian Gulf. Country boundaries are simplified and approximate; this figure is not intended for navigational or political-boundary use. Non-GCC neighboring countries (Iraq, Iran, Yemen) are shown in grey for orientation only and were not included in this review.

**Table 1:** Evidence gap map: availability of pediatric-onset multiple sclerosis data by country and domain.

Evidence Domain	Saudi Arabia	UAE	Kuwait	Qatar	Oman	Bahrain
Incidence/prevalence	+	+	+	–	–	–
Clinical phenotype	+	+	+	–	–	–
MRI/imaging phenotyping	–	±	–	–	–	–
DMT/treatment outcomes	±	+	+	–	–	–
Cognitive & quality-of-life outcomes	–	–	–	–	–	–
Biomarkers/genetics	–	–	–	–	–	–

+ = dedicated, pediatric-specific data reported. ± = partial or embedded data (e.g., pediatric subgroup within an adult cohort, or non-pediatric-specific imaging study including some pediatric onset cases). – = no peer-reviewed pediatric-specific data identified in this systematic search.

**Table 2:** Characteristics of identified primary studies reporting pediatric-onset multiple sclerosis data in the Gulf region.

Country	Study (First Author, Year)	Design/Data Source	Period	*n* (POMS)	Key Reported Findings
Dedicated POMS studies					
Kuwait	Alroughani et al., 2015 [8]	National MS Registry (est. 2011); retrospective, population-based	1994–2013	Registry-wide	Incidence 2.1/100,000 and prevalence 6.0/100,000 in 2013; significant annual rise in incidence (~5%) and prevalence (~6%), 1994–2013.
Kuwait	Alroughani et al., 2015 [9]	National MS Registry; retrospective comparison of pediatric- vs. adult-onset disease	Registry-wide	Not stated in abstract	Compared demographic/clinical features and disease course between pediatric- and adult-onset MS; informed prognostic indicators for secondary progression.
Kuwait	Alroughani et al., 2024 [10]	National MS Registry; retrospective cohort	Registry-wide, follow-up ≥ 10 y in 53% of cohort	249	Female: male ratio 2.4:1; mean age at onset ~15 years; brainstem/cerebellar (34.9%), spinal (29.3%) and visual (27.3%) presentations most common; 83.5% relapsing-remitting at last follow-up; annual relapse rate 0.18 in first 2 years on treatment.
Saudi Arabia	Bunyan et al., 2021 [11]	National Saudi MS Registry (2015–2018); multi-hospital, retrospective	2015–2018	287	Mean age at diagnosis 15.7 years; 74.2% female; hospital-registry prevalence 2.73/100,000; nationally projected prevalence 14.33/100,000 pediatric population.
UAE (Abu Dhabi)	Ismail et al., 2018 [12]	Multicenter, retrospective chart review, Emirati nationals	2010–2014	82	65% female; onset < 12 years presented predominantly with visual symptoms, onset ≥ 12 years with mixed visual/motor/sensory symptoms; age/sex-adjusted prevalence 26/100,000 (male) and 36/100,000 (female); incidence 2.3/100,000 (ages 10–14) and 7.2/100,000 (ages 15–19; this 15–19 band extends to age 19, beyond this review’s < 18-year POMS definition, and the 7.2/100,000 figure should be read as descriptive rather than a strict POMS incidence estimate (Section 3.3)); interferon beta-1a most common first-line DMT (48%).
UAE (Abu Dhabi)	Cleveland Clinic Abu Dhabi cohort, 2023 [13]	Single-center electronic health record review, retrospective	Not stated in abstract	103 (89 Emirati)	69.7% female; mean age at onset 15.0 years (range 8–17); 50.6% onset < 16 years; explored correlates of disease progression.
Pediatric DMT outcome studies					
Kuwait	Alroughani et al., 2017 [20]	National MS Registry; prospective, natalizumab-treated cohort	≥12 mo follow-up	32	72% female; mean age at onset 14.9 ± 2.6 y; 66% had breakthrough disease on first-line DMTs; annualized relapse rate reduced 1.66 → 0.06 (p < 0.001); EDSS improved 3.3 → 2.2 (p < 0.001).
Kuwait	Alroughani et al., 2023 [21]	National MS Registry; retrospective, ocrelizumab-treated cohort	Not stated in abstract	24	EDSS stabilized in 75% and improved in a further 16.7% of patients; MRI activity in only 4.2%; no evidence of disease activity (NEDA-3) achieved in 91.7%.
UAE (Abu Dhabi)	Altelly et al., 2023 [22]	Two-center (Cleveland Clinic Abu Dhabi & Sheikh Shakhbout Medical City) retrospective EMR review, ocrelizumab-treated cohort	Not stated in abstract	29	19 from CCAD, 10 from SSMC; 97% relapsing-remitting MS at treatment initiation; ocrelizumab reported as safe and well-tolerated in this uncontrolled cohort, with outcomes broadly consistent with international POMS and adult MS ocrelizumab literature (descriptive reported outcome, not comparative evidence of effectiveness).
Adult-inclusive studies (no dedicated pediatric subgroup)					
Qatar	Deleu et al., 2013 [23]	Adult-inclusive MS prevalence study (Hamad Medical Corporation), retrospective	Not fully stated	Not separately reported for pediatric onset	Crude MS prevalence 64.57/100,000; F:M 1.33:1. No dedicated pediatric/early-onset analysis reported; the previously cited “5% subgroup (*n* = 87)” could not be verified against this source.
Oman	Al-Senani et al., 2021 [24]	Hospital-based (Sultan Qaboos University Hospital & Khoula Hospital), retrospective, adult-inclusive	2006–2019 (prevalence); 2015–2018 (incidence)	Not separately reported for pediatric onset	Crude MS prevalence 15.9/100,000; annual incidence 1.38/100,000; mean age at onset 27.3 ± 7.7 years. No dedicated pediatric/early-onset subgroup analysis reported.

## Data Availability

No new data were created or analyzed in this study. Data sharing is not applicable to this article. The data-charting extraction spreadsheet will be made available via the Open Science Framework (OSF) upon publication.

## Supplementary Materials

The following supporting information can be downloaded at: https://www.mdpi.com/article/10.3390/biomedicines14102195/s1, Supplementary material, PRISMA-ScR checklist.

## Abbreviations

DMT	disease-modifying therapy
EDSS	Expanded Disability Status Scale
GCC	Gulf Cooperation Council
IPMSSG	International Pediatric Multiple Sclerosis Study Group
MS	multiple sclerosis
NEDA	no evidence of disease activity
POMS	pediatric-onset multiple sclerosis
PRISMA-ScR	Preferred Reporting Items for Systematic Reviews and Meta-Analyses extension for Scoping Reviews
UAE	United Arab Emirates
