# Supplementary material for: Pediatric-Onset Multiple Sclerosis in the Gulf: A Scoping Review of the Current Evidence Base and Research Gaps
Source: Biomedicines. 2026 Sep 28;14(10):2195. doi: 10.3390/biomedicines14102195 (PMC13701560; doi:10.3390/biomedicines14102195)
Supplement: Supplementary file 1 [file biomedicines-14-02195-s001.zip › biomedicines-4547817-supplementary.pdf]

SUPPLEMENTARY MATERIAL

PRISMA-ScR Checklist

Note: page references above correspond to the manuscript's internal section numbers rather than final typeset page numbers; authors should update to match paginated proof once assigned by the journal.

Section/Item	PRISMA-ScR Checklist Item	Reported on page #
**TITLE**		
Title	Identify the report as a scoping review.	Title page
**ABSTRACT**		
Structured summary	Provide a structured summary including background, objectives, methods, results, and conclusions.	Manuscript, Abstract
**INTRODUCTION**		
Rationale	Describe the rationale for the review in the context of what is already known.	Manuscript, Section 1
Objectives	Provide an explicit statement of the questions and objectives being addressed with reference to the Population-Concept-Context framework.	Manuscript, Section 1.1
**METHODS**		
Protocol	Indicate whether a review protocol exists; state if and where it can be accessed.	Manuscript, Section 2.1
Eligibility criteria	Specify characteristics of the sources of evidence used as eligibility criteria (e.g., years considered, language, publication status).	Manuscript, Section 2.2
Information sources	Describe all information sources in the search.	Manuscript, Section 2.3
Search	Present the full electronic search strategy for at least 1 database.	Manuscript, Section 2.3
Selection of sources of evidence	State the process for selecting sources of evidence.	Manuscript, Section 2.4
Data charting process	Describe the methods of charting data from the included sources.	Manuscript, Section 2.5
Data items	List and define all variables for which data were sought.	Manuscript, Section 2.5
Critical appraisal of individual sources	If done, provide a rationale and describe the method.	N/A (scoping review)
Synthesis of results	Describe the methods of handling and summarizing the data.	Manuscript, Section 2.6
**RESULTS**		

Selection of sources of evidence	Give numbers of sources screened, assessed for eligibility, and included.	Manuscript, Section 3.1; Figure 1
Characteristics of sources of evidence	For each source, provide characteristics and citation details.	Table 1
Critical appraisal within sources	If done, provide data on critical appraisal.	N/A (scoping review)
Results of individual sources	For each source, present the relevant data that were charted.	Table 1; Manuscript, Sections 3.2-3.7
Synthesis of results	Present the main results of the review.	Manuscript, Sections 3.2-3.8; Table 2
**DISCUSSION**		
Summary of evidence	Summarize the main results, including any limitations.	Manuscript, Section 4
Limitations	Discuss limitations of the scoping review process.	Manuscript, Section 5
Conclusions	Provide a general interpretation of the results.	Manuscript, Section 6
**FUNDING**		
Funding	Describe sources of funding for the included sources of evidence and the review itself.	Manuscript, Declarations
